# Supplementary material for: DMRforPairs: identifying Differentially Methylated Regions between unique samples using array based methylation profiles
Source: BMC Bioinformatics. 2014 May 15;15:141. doi: 10.1186/1471-2105-15-141 (PMC4046028; doi:10.1186/1471-2105-15-141)
Supplement: Additional file 2 — DMRforPairs output for the comparison of A431-MCF7 and NA17018-NA17105. Please start from the HTML files in each folder. Available via the BMC Bioinformatics website. [file 1471-2105-15-141-S2.zip › 1394847754114233_MOESM2_ESM/A431_MCF7/figures/17637.pdf]

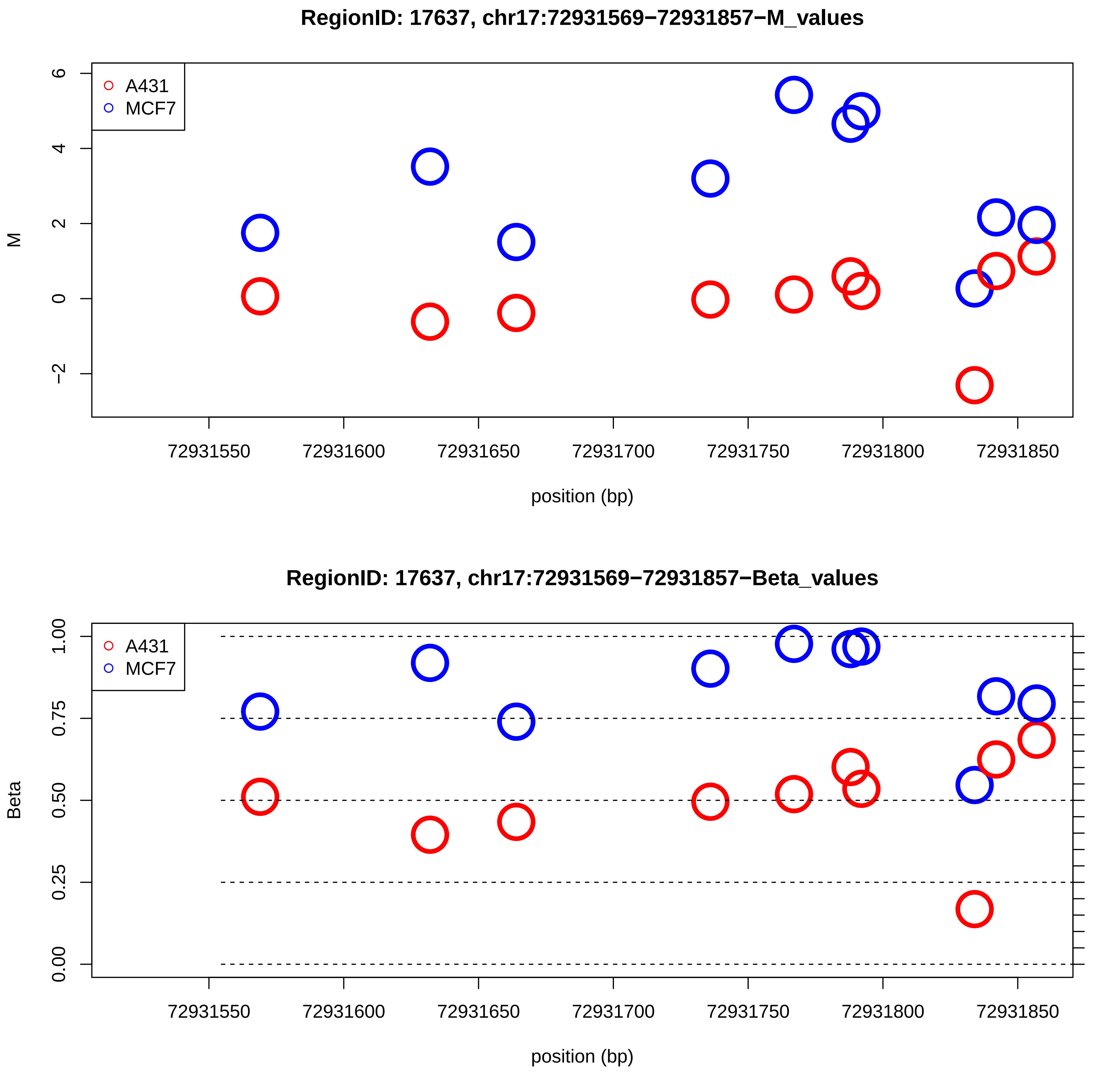

RegionID: 17637, chr17:72931569−72931857−M_values
A431
MCF7
M
6
4
2
0
−2
72931550
72931600
72931650
72931700
72931750
72931800
72931850
position (bp)
RegionID: 17637, chr17:72931569−72931857−Beta_values
A431
MCF7
Beta
1.00
0.75
0.50
0.25
0.00
72931550
72931600
72931650
72931700
72931750
72931800
72931850
position (bp)

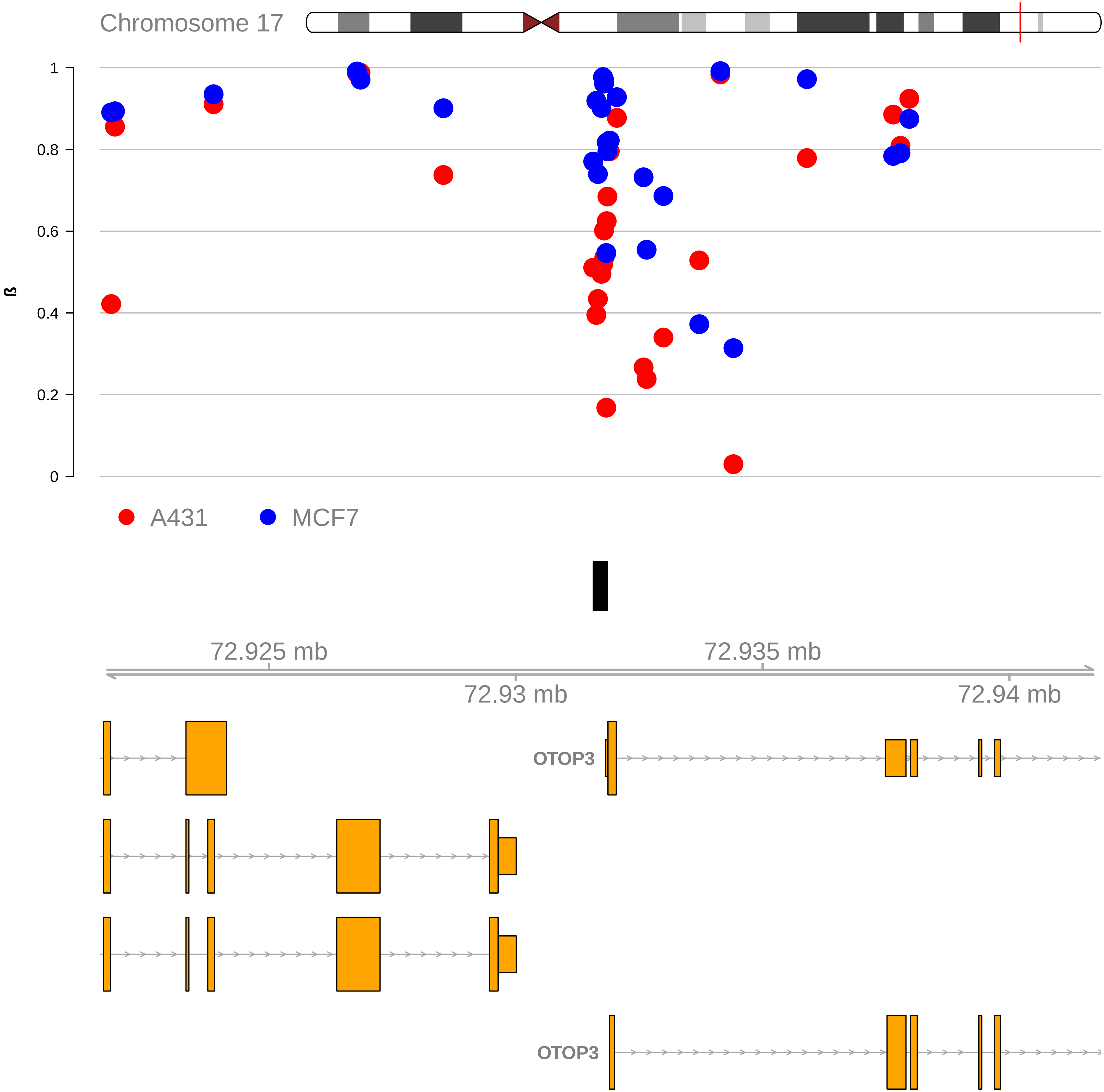

Chromosome 17
ß
1
0.8
0.6
0.4
0.2
0
A431
MCF7
72.925 mb
72.935 mb
72.93 mb
72.94 mb
OTOP3
OTOP3
